# 141 Focused Contact Precautions and Data-Driven Dashboards to Improve MRSA Hospital Acquired Infection Rates at the University of Kentucky

**DOI:** 10.1017/ash.2026.10721

**Published:** 2026-06-23

**Authors:** Daniel Muleta, Carissa Wallace, Sarah Petersen, Olivia Denzie, Christopher Wilson, Becky Meyer

**Affiliations:** 1 Tennessee Department of Health

## Abstract

**Introduction** Carbapenem Resistant Acinetobacter Baumannii complex (CRAB) is classified as an urgent public health threat by the Centers for Disease Control and Prevention (CDC). Tennessee conducts population-based CRAB surveillance collaborating with CDC as part of the Emerging Infections Program (EIP). Methods A case was defined as Acinetobacter baumannii complex isolated from normally sterile sites, urine, lower respiratory tract specimens or wound specimens resistant to meropenem, imipenem or doripenem from a resident of the surveillance area. Polymerase chain reaction (PCR) testing was performed for gene detection on the available isolates submitted to the state public health lab since 2018. Epidemiologic and clinical data was collected by reviewing the medical records. A chi-square test was run comparing categorical variables, and logistic regression was done comparing odds ratios controlling for potential confounders. Data analysis was done using SAS 9.4 Results From 2014–2024, 249 cases were identified from the surveillance region. Mean age among cases was 60.9 years. There were 98(39.4%) females, 167 (67.1%) white persons, 77 (30.9%) African Americans and 5 (2.0%) other races. Carbapenemase-producing (CP) genes were detected from 108 (82.4%) of 131 available isolates tested at the state public health lab. The proportion of isolates with CP-genes increased to 85.7% and above since 2020, compared to the rate in 2019 (35%) (P<0.001). OXA-24/40, OXA-51, and OXA-23 were identified from 44.6%, 35.8%, and 19.6% of tested isolates, respectively. Additionally, 38.8% of these positive isolates had multiple gene types. OXA-23 was the most prevalent gene until 2020 (53.8%). OXA-24/40 and OXA-51 have become predominant since 2021, accounting for 44.4% and 35.8% of all reported cases, respectively. Hospital admission rates among patients with invasive infection were 10-fold higher (OR=10.03, P= 0.025) compared to non-invasive. The average length of hospital stay was 17 days. Most of the cases (93.1%) were healthcare-associated infections, and there was no significant change in the proportion of community-associated infections across the years (P=0.289) Conclusion The proportion of isolates with CP-gene has increased over the years. Most of the infections required hospital admission with longer hospital stay compared to the state average of 4.84 days, suggesting the infection is associated with higher medical costs and severe infection. The findings from this surveillance analysis assert the importance of infection prevention in healthcare settings as CP-CRABs are increasing. Monitoring the trends of CP-genes is vital as the types of CP-genes change over time.

